# QUantitative and Automatic Atmospheric Correction (QUAAC): Application and Validation

**DOI:** 10.3390/s22093280

**Published:** 2022-04-25

**Authors:** Shumin Liu, Yunli Zhang, Limin Zhao, Xingfeng Chen, Ruoxuan Zhou, Fengjie Zheng, Zhiliang Li, Jiaguo Li, Hang Yang, Huafu Li, Jian Yang, Hailiang Gao, Xingfa Gu

**Affiliations:** 1 School of Software Engineering, Jiangxi University of Science and Technology, Nanchang 330013, China; liushumin@jxust.edu.cn (S.L.); 6720200908@mail.jxust.edu.cn (Y.Z.); zhouruoxuan@jxust.edu.cn (R.Z.); lijg@aircas.ac.cn (J.L.); 2 Aerospace Information Research Institute, Chinese Academy of Sciences, Beijing 100101, China; zhaolm@aircas.ac.cn (L.Z.); yanghang@aircas.ac.cn (H.Y.); lihf@aircas.ac.cn (H.L.); yangjian@aircas.ac.cn (J.Y.); gaohl200439@aircas.ac.cn (H.G.); guxf@aircas.ac.cn (X.G.); 3 School of Space Information, Space Engineering University, Beijing 101416, China; zhengfj@radi.ac.cn (F.Z.); lizhiliang_1988@foxmail.com (Z.L.); 4 University of Chinese Academy of Sciences, Beijing 100049, China; 5 School of Remote Sensing and Information Engineering, North China Institute of Aerospace Engineering, Langfang 065000, China

**Keywords:** atmospheric correction, radiative transfer model, aerosol optical thickness, surface reflectance, FLAASH

## Abstract

The difficulty of atmospheric correction based on a radiative transfer model lies in the acquisition of synchronized atmospheric parameters, especially the aerosol optical depth (AOD). At the moment, there is no fully automatic and high-efficiency atmospheric correction method to make full use of the advantages of geostationary meteorological satellites in large-scale and efficient atmospheric monitoring. Therefore, a QUantitative and Automatic Atmospheric Correction (QUAAC) method is proposed which can efficiently correct high-spatial-resolution (HSR) satellite images. QUAAC uses the atmospheric aerosol products of geostationary satellites to match the synchronized AOD according to the temporal and spatial information of HSR satellite images. This method solves the problem that the AOD is difficult to obtain or the accuracy is not high enough to meet the demand of atmospheric correction. By using the obtained atmospheric parameters, atmospheric correction is performed to obtain the surface reflectance (SR). The whole process can achieve fully automatic operation without manual intervention. After QUAAC applied to Gaofen-2 (GF-2) HSR satellite and Himawari-8 (H-8) geostationary satellite, the results show that the effect of QUAAC correction is slightly better than that of the Fast Line-of-sight Atmospheric Analysis of Spectral Hypercubes (FLAASH) correction, and the QUAAC−corrected surface spectral curves have good coherence to that of the synchronously measured by field experiments.

## 1. Introduction

The remote sensing images taken by satellite are easily affected by the atmosphere, which interfere with obtaining the actual surface reflectance (SR) of the target objects. Most remote sensing applications rely on SR products [1], but acquiring SR is still a challenge [2,3]. Therefore, in order to improve the quality of images and restore the actual SR of target objects, it is necessary to eliminate the top-of-atmosphere (TOA) contributions from atmospheric molecules, aerosols, and other atmospheric components on remote sensing images through atmospheric correction.

There are many methods of atmospheric correction, which are mainly divided into two methods which are respectively based on images and physical models [4]. The image-based method is relatively simple, and only inverts the reflectivity by the image data, such as dark object subtraction (DOS) [5] and the empirical line method (ELM) [6]. The physical model is mainly based on radiative transfer model (RTM)—such as the second simulation of the satellite signal in the solar spectrum (6S) [7]—and the moderate resolution atmospheric transmission (MODTRAN) [8] model [9]. Under the same conditions, the physical correction method based on the RTM is relatively more accurate than the image-based method [10]. However, the difficulty lies in the acquiring of synchronized atmospheric parameters, especially aerosol optical depth (AOD). The distribution of aerosols in the atmosphere varies greatly with time and space, which makes it difficult to obtain matched inputs for atmospheric correction [11]. The previous aerosol data retrieved by old generation geostationary satellites have low accuracy due to the lack of aerosol sensitive spectral bands [12], even if they have a very high temporal resolution.

Shekhar et al. [13] proposed a Flexible Atmospheric Compensation Technique (FACT) method to correct the hyperspectral and multispectral remote sensing data by simulating the outputs of 6S RTM with various inputs. Compared with the Fast Line-of-sight Atmospheric Analysis of Spectral Hypercubes (FLAASH) correction, the accuracy of the FACT was about 95% for hyperspectral imaging sensors and close to 98% for multispectral imaging sensors. Wang et al. [14] proposed a coupling parameter based atmospheric correction (CPBAC) method to correct the Landsat 7 multispectral data by calculating the coupling parameters of atmosphere and topography, and the corrected result was similar to that produced by FLAASH. However, CPBAC can only be used for data containing good Lambert objects under variant topography, which limited its widespread application. Katkovsky et al. [15] proposed a new algorithm for atmospheric correction of hyperspectral images which used the atmospheric parameters and the average SR to calculate the spectral reflectance of all other pixels under the assumption of a horizontal homogeneity of the atmosphere within the image. The spectra corrected by this algorithm had good correspondence with the referenced SR. The above methods have many limitations, for example the atmospheric parameters are difficult to obtain.

Cao et al. [16] corrected Gaofen-2 (GF-2) high-spatial-resolution (HSR) satellite multispectral data with AOD retrieved from the moderate-resolution imaging spectroradiometer (MODIS) data. Compared with the relative error of reflectance before atmospheric correction, the difference between the corrected SR and the measured reflectance was obviously narrowed. David et al. [17] proposed a MODIS-based method that used AOD, aerosol type and water vapor from MODIS Terra to correct Landsat Enhanced Thematic Mapper Plus (ETM+) acquisitions in each coincident orbit. The performance of the MODIS-based atmospheric correction is better than that of the image-based the Landsat Ecosystem Disturbance Adaptive Processing System (LEDAPS) method. Basith et al. [18] used the AOD parameter which was retrieved from SR inversion involving daily global SR products of MODIS, to corrected Landsat-8 images based the 6S method. The results of atmospherically corrected images were agreeable with the Landsat 8 Level-2 products. However, the satellite with a Sun-synchronous orbit cannot provide a high-temporal AOD, which is hard to match the HSR satellite data such as GF-2. More and more new generation geostationary meteorological satellites have been launched, such as Himawari-8 (H-8) [19], Fengyun-4 [20]. Wang et al. [21] conducted work dedicated to exploiting information from the diurnal variability in the hypertemporal geostationary observations for atmospheric correction, and the result of the algorithm correction agreed well with the ground-based measurements. The method shows that the high-temporal-resolution observation information can help to address the atmospheric correction problem. It is necessary to propose a fully automatic and efficient atmospheric correction method for HSR satellite images, taking advantage of atmospheric products provided by geostationary meteorological satellites [22].

In this paper, a QUantitative and Automatic Atmospheric Correction (QUAAC) method is proposed, which is fast running and accurate. This method makes full use of the advantages of geostationary meteorological satellites’ atmospheric monitoring to solve the problem of obtaining atmospheric data. Atmospheric data from geostationary satellite observations are matched according to the spatial and temporal information consistent with the HRS satellite data. Then, an atmospheric correction based on RTM is carried out to obtain the SR of HSR satellite images. The QUAAC algorithm achieves a complete processing chain of atmospheric correction without manual operation. The QUAAC method is applied to GF-2 HSR satellite and H-8 geostationary satellite and its accuracy was verified.

## 2. Materials and Methods

### 2.1. Digital Elevation Model

A digital elevation model (DEM) is a representation of the bare ground (bare earth) topographic surface of the Earth excluding surface objects such as trees or buildings, and is a quantitative representation of terrain [23]. DEM is the high-resolution raster data that covers the global DEM value in numerical form. Each pixel value on the DEM represents the DEM value of the geographic location. The DEM provides important remote sensing data for atmospheric RTM [24] which are used to obtain the DEM value in different geographic locations. A DEM is used which is extracted from Shuttle Radar Topography Mission (SRTM) with a 90 m spatial resolution [25]. In the first version, the easily accessible SRTM with a 90 m spatial resolution was used. In future improved versions, the DEM will be updated with a higher resolution (e.g., 30 m).

### 2.2. GF-2 Satellite Data

The GF-2 satellite is the first civil optical remote sensing satellite with spatial resolution better than 1 m developed by China. It was successfully launched at the Taiyuan Satellite Launch Center on 19 August 2014. The spatial resolution of the sub-satellite point can reach 0.8 m [26]. The GF-2 satellite is equipped with two high-resolution cameras of 1 m panchromatic and 4 m multispectral images [27], and contains four bands of blue, green, red, and near-infrared [28] as shown in Table 1.

The radiometric calibration coefficient and the spectral response function of the GF-2 satellite is used for atmospheric correction, and they can be obtained from The China Centre for Resource Satellite Data and Application (CRESDA, http://www.cresda.com/CN/, accessed on 5 April 2022). Every year, the calibration coefficient is published by CRESDA from field calibration experiments, and the accuracy of the calibration coefficient is better than 5.3% [29].

### 2.3. Aerosol Products from Himawari-8 Satellite

The H-8 satellite is a new generation of geostationary meteorological satellite from the Japan Aerospace Exploration Agency (JAXA). It was successfully launched in Tanegashima, Japan on 7 October 2014, and has been measuring officially since 7 July 2015 [30,31]. The Advanced Himawari Imager (AHI) onboard H-8 can achieve an Earth observation every 10 min, which acquires data from 0.47 to 13.3 µm in 16 spectral bands [32]. The monitoring area of AHI is 60° N–60° S, 80° E–160° W, covering most of the western North Pacific. AHI provides images with a spatial resolution down to 500 m every 10 min (fulldisk) [33].

In this study, the AHI level-2 aerosol products (ARP) were downloaded to use from the JAXA “P-Tree” system (ftp://ftp.ptree.jaxa.jp, accessed on 7 April 2022). ARP’s four datasets—longitude, latitude, AOT and AOT_uncertainty—are matched to obtain the synchronized AOD of GF-2 satellite data. The value of AOT dataset represents AOD in this APR data. The value of AOT_uncertainty dataset represents the degree of AOT uncertainty. The AOT_uncertainty dataset is divided into three parts according to the value, and different value ranges represent different AOT dataset’s confidence levels as listed in Table 2. The retrieval accuracy of the H-8 ARP is generally good [34,35], controlling quality by the AOT_uncertainty dataset is helpful for identifying AOD accuracy.

### 2.4. QUAAC Algorithm

**Figure 1:**
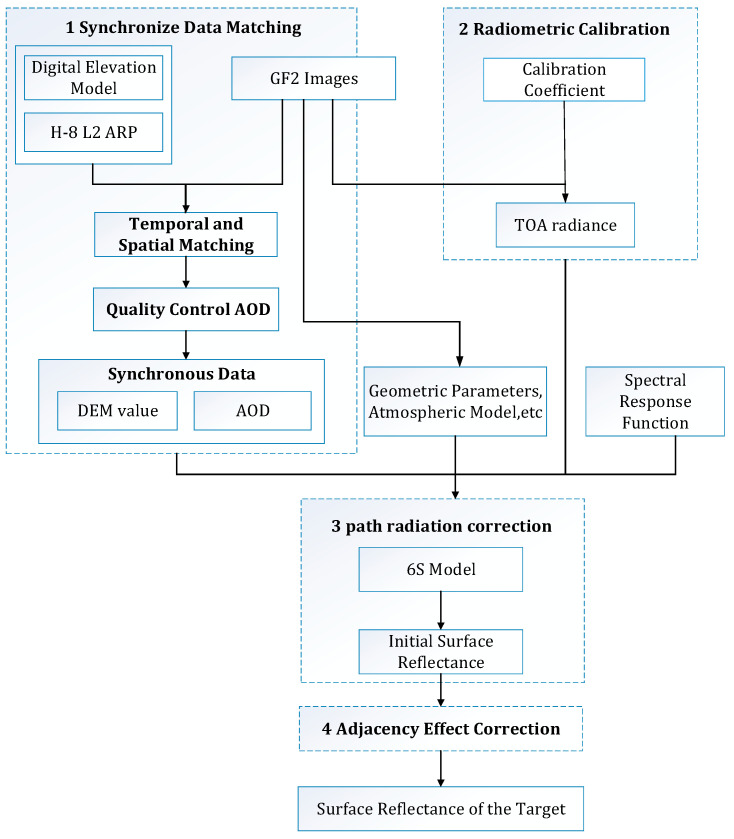
Flowchart of the QUAAC algorithm.

For the atmospheric correction of HSR satellites, we propose a method for atmospheric correction based on 6S RTM, using the atmospheric aerosol products observed by geostationary satellites. The algorithm is applied to GF-2 satellite image and H-8 Satellite level 2 ARP as shown in Figure 1.

In the first step, the pixels of the same spatial data—as GF-2 satellite image in the DEM—are matched, and the AOT and AOT_uncertainty datasets’ pixels of the same temporal (no more than 5 min difference) and spatial data as GF-2 satellite images in the H-8 Satellite level 2 ARP are matched. If the matched pixel value of the AOT_uncertainty dataset is greater than 0.5, the AOT dataset’s pixel value in the AOT_uncertainty dataset is discarded. After data matching and quality control, the synchronized DEM value and AOD data are obtained.

In the second step, it is necessary to perform radiometric calibration for GF-2 satellite image by absolute radiometric calibration coefficient. Radiometric calibration is employed to convert the digital number (DN) of the original remote sensing images into TOA radiance [16]. The definition of radiometric calibration is

(1) L=offset+Gain×DN 

where L is the TOA radiance after radiometric calibration, offset is the offset of absolute calibration coefficient, and Gain is the gain value.

The formula for calculating TOA reflectance $\rho_{TOA}$ is

(2) $$\rho_{TOA}=\frac{\pi Ld^{2}}{cos\left(\theta \right)ESUN_{\lambda }}$$

where *d* is the astronomical distance from the Sun to the Earth, θ is the zenith angle of the Sun, and $ESUN_{\lambda }$ is the solar spectral irradiance at the upper boundary of the atmosphere with the central wavelength of *λ*.

In the third step, the solar and satellite observation geometry, observation date, and atmospheric model are obtained by the GF-2 images data. There are the following atmospheric models: tropical, mid-latitude summer, mid-latitude winter, sub-arctic summer, and sub-arctic winter. The atmospheric model is defined by the time and location of GF-2 images. The above data are input into 6S model together with spectral response function, synchronized DEM value, and AOD. Then, the atmospheric correction coefficients are output to complete the path radiometric correction. In this way, the influence of atmospheric molecules on the target objects is eliminated. The initial SR is calculated with the radiance L. The calculation method of the initial SR as

(3) $$\rho_{ARC}=\frac{X_{a}L-X_{b}}{1+\left(X_{a}L-X_{b}\right)X_{c}}$$

where $X_{a}$, $X_{b}$, and $X_{c}$ are correction coefficients from outputs of 6S RTM, and $\rho_{ARC}$ are initial SR.

Finally, it is significant to eliminate the influence caused by the adjacent pixels around the target pixel on the target pixel [16]. The TOA radiance $\rho_{TOA}$ is composed of path radiation reflectance $\rho_{a}$ from 6S output, target pixel reflectance $\rho_{t}$, and background reflectance $\rho_{b}$. The expression is

(4) $$\rho_{TOA}=\rho_{a}+T_{\left(\theta_{s}\right)}T_{\left(\theta_{\nu }\right)}\left[\rho_{t}\iint PSF_{tar}\left(x,y\right)\;dxdy+\rho_{b}\iint PSF_{back}\left(x,y\right)\;dxdy\;\right]\;$$

where $T_{\left(\theta_{s}\right)}$ and $T_{\left(\theta_{\nu }\right)}\;$are the downward and upward radiative transmittance respectively from outputs of 6S RTM, where $PSF_{tar}$ is atmospheric point spread function for the target pixel, and $PSF_{back}$ is atmospheric point spread function for the background pixel.

The formula for background reflectance $\rho_{b}$ is

(5) $$\rho_{b}\left(x,y\right)=\frac{\sum_{i=-n}^{n}\sum_{j=-n}^{n}\rho_{ARC}\left(i,j\right)e^{-r}}{\sum_{i=-n}^{n}\sum_{j=-n}^{n}e^{-r}}$$

where r is actual distance of the reference pixel and the central pixel.

The r is calculated as

(6) $$r=a\sqrt{i^{2}+j^{2}}\;$$

where a is spatial resolution of the GF-2 satellite image.

Over an entire area, if ∬PSF(x,y) dxdy=1, $\iint PSF_{tar}\left(x,y\right)\;dxdy=\alpha$ (0 < α < 1) and $\iint \;PSF_{back}\left(x,y\right)\;dxdy=1-\alpha$.

Equation (4) can be

(7) $$\rho_{TOA}=\rho_{a}+T_{\left(\theta_{\nu }\right)}T_{\left(\theta_{s}\right)}\left(\rho_{t}\alpha +\rho_{b}\left(1-\alpha \right)\right)$$

where α is the contribution rate of target pixel’s actual SR $\rho_{t}$ to $\rho_{ARC}$.

The α reflects the extent of adjacency effect. After removing $\rho_{a}$ and attenuation of Equation (7), $\rho_{ARC}$ is obtained as

(8) $$\rho_{ARC}=\rho_{t}\alpha +\rho_{b}\left(1-\alpha \right)$$

Then, the SR after the adjacency effect correction is obtained as

(9) $$\rho_{t}=\frac{\rho_{ARC}-\rho_{b}\left(1-\alpha \right)}{\alpha }$$

The adjacency effect is corrected via a long computational period. The parallel computing is a solution method for the temporal acceleration. The parallel strategy uses multi-CPU to work simultaneously for atmospheric correction of GF-2 image [36]. When the CPU number increases, the computational speed also increases. Under the 8-CPU working mode, a multispectral image atmospheric correction only takes 1 min.

### 2.5. QUAAC Validation

#### 2.5.1. Measured Surface Reflectance (MSR) Data

**Figure 2:**
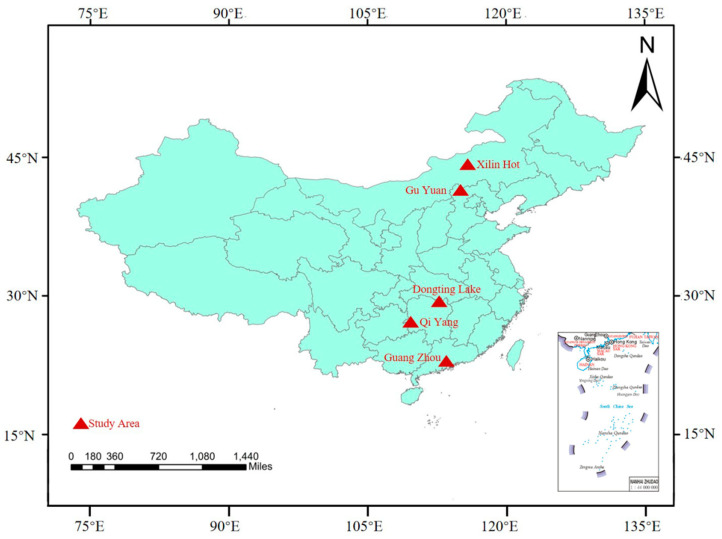
Five test sites for surface spectral reflectance measurement. The areas marked in red on the map are the test areas—Xilinhot, Guyuan, Dongting Lake, Qiyang, and Guangzhou—from top to bottom.

To verify the accuracy of QUAAC, the multispectral images of the GF-2 satellite were downloaded as HSR data, and five test sites (Dongting Lake, Guyuan, Qiyang, Guangzhou, and Xilinhot) were selected as shown in Figure 2. In Dongting Lake, Guyuan, Qiyang, and Guangzhou, there are various types of land objects, and grass is very abundant in Xilinhot. The specific information of the multispectral images is shown in Table 3. The field spectral data of surface types measured at the same time and in the sites are obtained (http://nsicat.radi.ac.cn, accessed on 29 March 2022). The MSR values were collected in raw DN mode with an ASD FieldSpec 4 spectroradiometer with the 8° foreoptic attachment. The spectrometer has a 400–2500 nm wavelength range, and the 450–900 nm wavelength range is used in this study. The uncertainty of the spectrometer measurement is no more than 3% in 450–900 nm [37].

In order to obtain an accurate MSR, the measured spectral curve of the field objects and the spectral response function of the GF-2 satellite are convolutionally calculated as

(10) $$MSR=\int_{a}^{b}f\left(\tau \right)g\left(x-\tau \right)d\tau$$

where a and *b* are the band range, and *f(x)* and *g(x)* respectively represent the measured spectral curve and spectral response function.

#### 2.5.2. Statistical Index

QUAAC validation is mainly based on MSR. The statistical parameters of remote sensing image quality can be divided into two categories according to the number of factors involved in the evaluation [38]. One is single factor statistical indexes—including entropy values, standard deviation, average gradient, etc. The other is comprehensive statistical indexes—including root mean square error (RMSE), correlation coefficient (R), etc.

In order to test the effect of QUAAC correction, this study selects normalized difference vegetation index (NDVI) [39], information entropy (IE), and average gradient (AG) [40] as the quality statistical index of single image. The RMSE [13], relative error (RE) [41], mean absolute error (MAE) [34], R, and coefficient of determination ($R^{2}$) [42] are used as comprehensive statistical indexes.

NDVI can reflect the growth status and nutrition information of green vegetation. The larger the value of NDVI, the greater the amount of green vegetation coverage. The IE represents the amount of information provided by image. AG reflects the rate of change in the contrast of tiny details of the image, indicating the relative clarity of the image. The larger the AG is, the higher the clarity of the image. RMSE describes the degree of deviation between data. The smaller the RMSE, the smaller the degree of deviation. The RE can reflect the reliability of the predicted value. The MAE represents the average value of the absolute error between the predicted value and the observed value, reflecting the size of the actual prediction error. The R represents the correlation between data, the closer the R value is to 1, the higher the similarity between the two sets of spectral curves. The larger the $R^{2}$, the greater the goodness of fit.

## 3. Results and Discussion

### 3.1. Image Quality Evaluation

**Figure 3:**
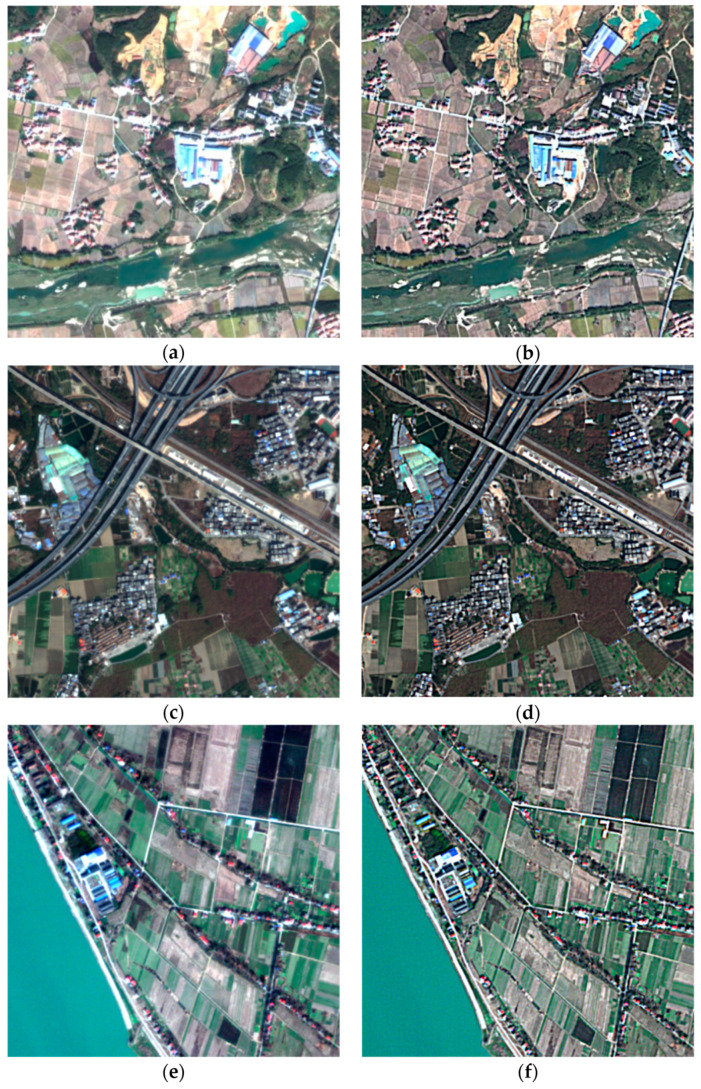

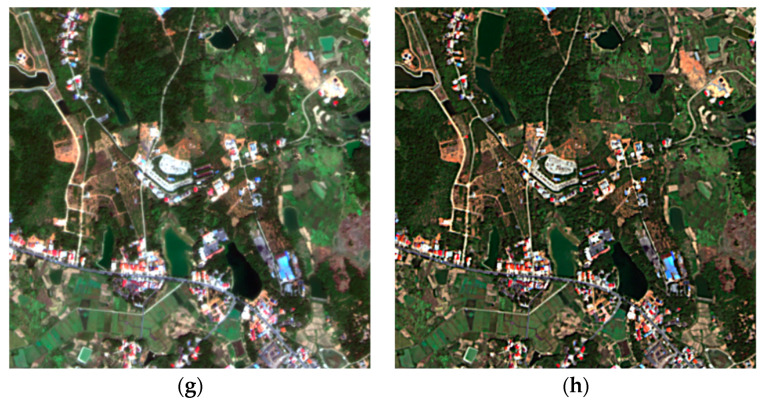
Comparison of GF-2 images before and after QUAAC correction. True color composite images of TOA Radiance are shown on the left column and QUAAC−correcteded SR images are shown on the right column. The dates and locations from top to bottom are Dongting Lake on 11 November 2020 (**a**,**b**), Guangzhou on 29 January 2021 (**c**,**d**), Dongting Lake on 15 January 2021 (**e**,**f**), and Qiyang on 6 July 2021 (**g**,**h**).

Sixteen GF-2 multispectral images were corrected by QUAAC. The visual comparison between the images before and after QUAAC correction is shown in Figure 3. It is found that after QUAAC, the clarity and contrast of the images are visually improved.

The IE and AG of the TOA radiance image and the QUAAC−corrected SR images were calculated to evaluate the quality of the images as shown in Table 4. The data show that after QUAAC and FLAASH correction, the IE and AG of images increase significantly, which indicates that the informational content and clarity of the corrected images are improved. Most of the IE of FLAASH is higher than that of QUAAC, indicating that the amount of information contained in images after FLAASH correction is slightly better than that of QUAAC correction. The average gradient of QUAAC is significantly higher than that of FLAASH, indicating that the clarity of QUAAC correction is better than that of FLAASH.

**Figure 4:**
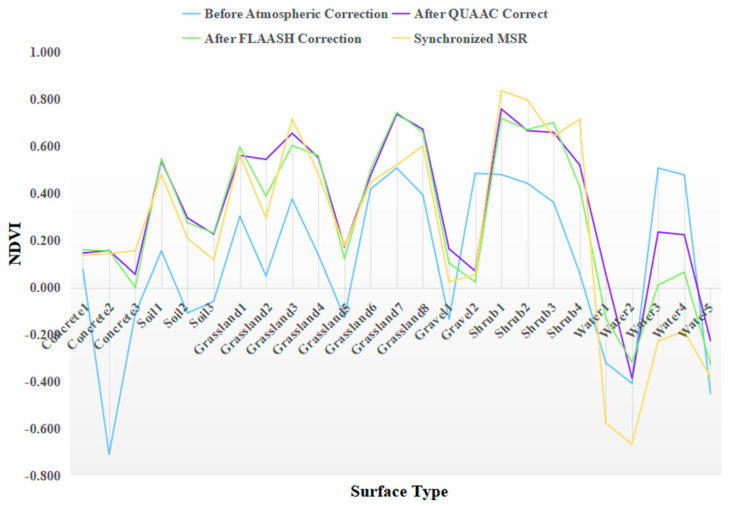
NDVIs of concrete floor, soil, grassland, gravel, shrubs, and water before and after QUAAC and FLAASH correction and synchronized MSR. The blue represents the NDVI before atmospheric correction, the purple represents the QUAAC−corrected NDVI, the green represents the FLAASH−corrected NDVI, and the orange represents the NDVI of synchronized MSR.

In this experiment, the following typical surface types were also selected as samples: concrete floor, soil, grassland, gravel, shrub, and water. The NDVI of these objects is calculated, and the NDVI of the surface types before and after QUAAC correction is compared as shown in Figure 4. The results indicate that the NDVI of all surface types changes, and the NDVI of corrected green vegetation is significantly increased. The NDVI curves of QUAAC correction, FLAASH correction, and MSR involve many overlapping parts, and numerical gaps are small. This shows that QUAAC improves the ability of extracting green vegetation information, which is beneficial to the distinction between green plants and other surface types.

### 3.2. Validation of Spectral Reflectance on Different Surface Types

**Figure 5:**
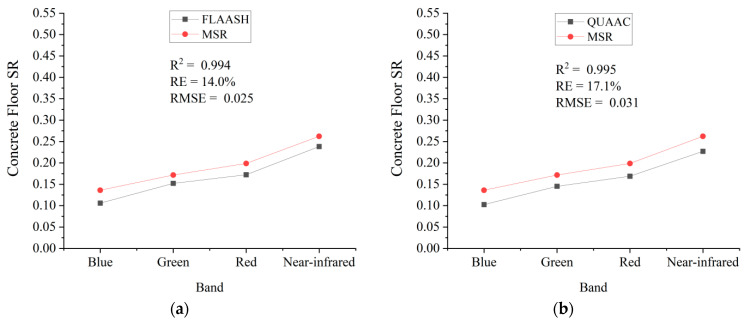

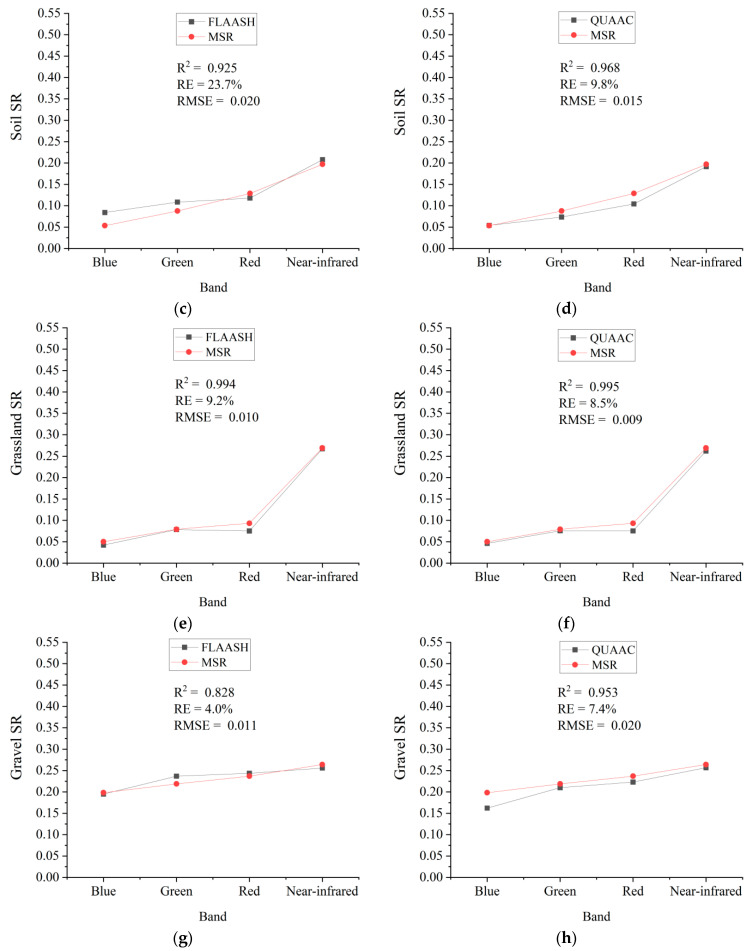

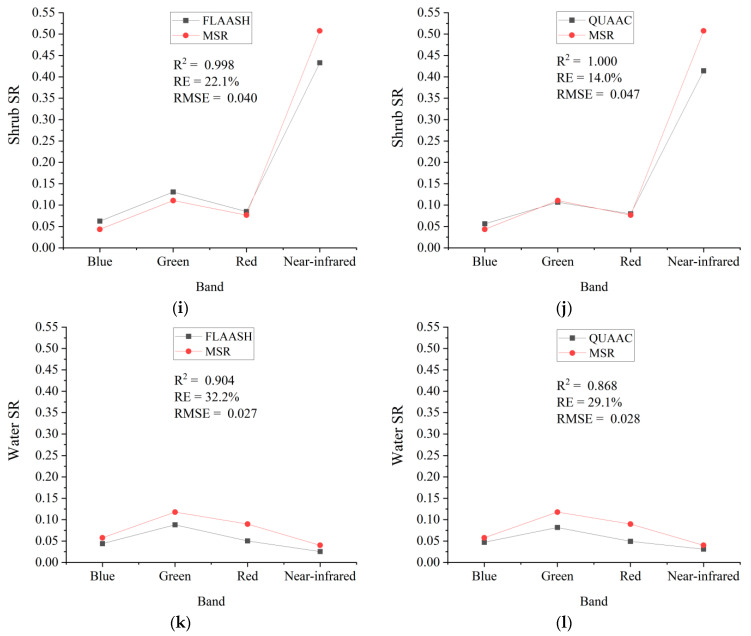
Comparison of QUAAC (right column) and FLAASH (left column) corrected spectral reflectances versus synchronized MSR on each surface type. From top to bottom, they are the concrete floor of Dongting Lake on 11 November 2020 (**a**,**b**), the soil of Dongting Lake on 15 January 2021 (**c**,**d**), the grassland of Xilinhot on 27 June 2020 (**e**,**f**), the gravel of Qiyang on 3 September 2021 (**g**,**h**), the shrub of Qiyang on 6 July and 2021 (**i**,**j**), the water of Guangzhou on 29 January 2021 (**k**,**l**). Each subfigure is marked with $R^{2}$, RE, and RMS.

The synchronized MSR is used as the reference data. Multispectral images on the same date and location after FLAASH and QUAAC correction are selected. We compare the FLAASH/QUAAC−corrected SR of concrete floor, soil, grassland, gravel, shrub, and water with the synchronized MSR respectively. The${\;R}^{2}$, RE, and RMSE are used to evaluate the accuracy. The comparison charts are shown in Figure 5.

Figure 5 shows that six surface types’ spectral curve trends of FLAASH/QUAAC correction are basically the same as those of MSR. The QUAAC−corrected effects of soil, grassland, and shrub are slightly better than those of FLAASH; and the FLAASH−corrected effects of concrete floor, gravel, water are slightly better than those of QUAAC. After QUAAC, the corrected effects of surface types are ranked from good to bad, followed by grass, shrubs, soil, gravel, concrete floor, and water. The deviation between SR of QUAAC−corrected grassland and synchronized MSR is smallest, and the similarity is high.

The reflectance of water in each band is low, and the errors are brought by factors such as atmosphere, AOD, and radiation calibration coefficient. This will lead to relative errors, resulting in a large deviation between thewater reflectance after QUAAC correction and the synchronized MSR. The $R^{2}$ of concrete floor is high, and the trends of the spectral curves are the same. However, the concrete floor’s SR of each band has a systematic deviation. Maybe it is because validation pixels in the GF-2 image are mixed-pixel, and the field measured data are a single-point measurements, resulting in systematic errors.

### 3.3. Validation on Different Spectral Bands

**Figure 6:**
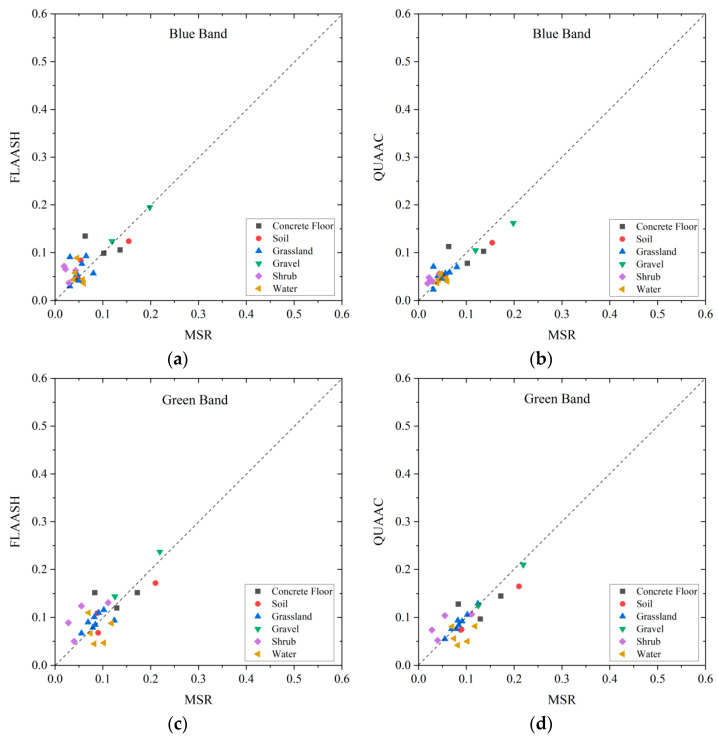

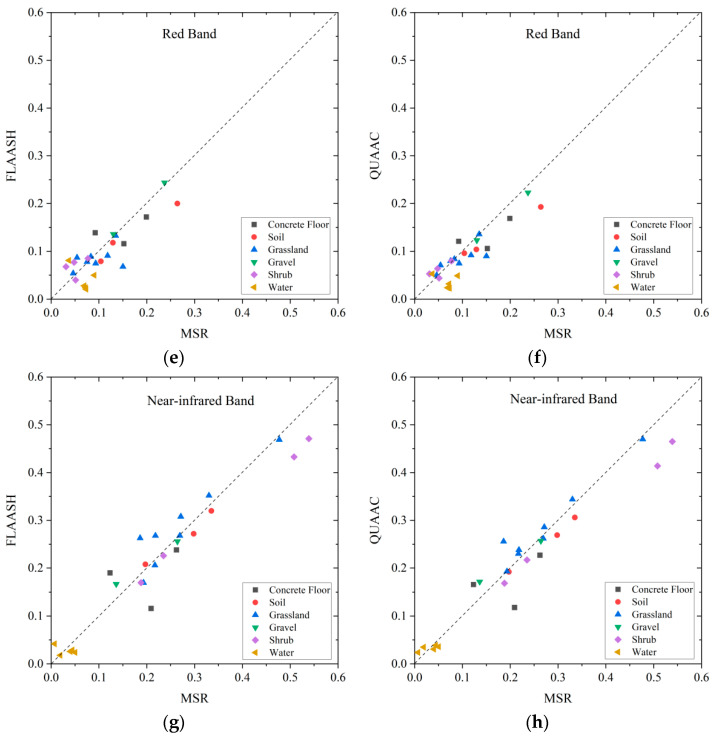
Scatter plots of QUAAC (right column) and FLAASH (left column) corrected SR versus MSR in each band. From top to bottom, the four bands are blue (**a**,**b**), green (**c**,**d**), red (**e**,**f**), and near-infrared (**g**,**h**). Different shapes and colors in the figure represent different surface types.

In order to better analyze the corrected effect of QUAAC on each band, the QUAAC−corrected SR and the synchronized MSR, and the FLAASH−corrected SR and the synchronized MSR in the blue, green, red, and near-infrared bands are compared as shown in Figure 6. The closer the point is to the diagonal, the smaller the gap between the FLAASH/QUAAC−corrected reflectance and the synchronized MSR. Respectively, the MAE, RMSE, $R^{2}$, and R between the FLAASH/QUAAC−corrected reflectance and the synchronized MSR are calculated to evaluate the accuracy as listed in Table 4.

The scatter diagram in Figure 5 shows that the surface types in the four bands are distributed around the diagonal as a whole. In the blue, green, and red bands, the SR are basically below 0.25, and in the near-infrared band, the SR spans are larger, from 0 to 0.6. The statistical index of FLAASH, QUAAC, and MSR as listed in Table 5 show that QUAAC is better than FLAASH in each index in each band. The QUAAC−corrected effect is the best in the near-infrared band. Probably because the QUAAC computes the atmospheric radiative transfer more correctly. Generally, the QUAAC−corrected SR and synchronized MSR have a small deviation, high similarity, and consistent spectral trend.

## 4. Conclusions

In this study, QUAAC uses the atmospheric aerosol products observed by H-8 satellite to provide synchronized data with the GF-2 satellite images, and successfully achieves automatically atmospheric correction of the GF-2 images based on the 6S RTM. The images before and after QUAAC correction were compared, and six kinds of surface types including concrete floor, soil, grassland, gravel, shrub, and water are selected to verify the QUAAC accuracy. The QUAAC-correcteded and FLAASH−corrected spectral curves were compared with synchronized MSR. The results show that the NDVI and clarity after QUAAC correction are significantly increased. After horizontal comparison, the corrected effect of QUAAC is slightly better than that of FLAASH. The spectral curve trend of the surface types is basically the same as that of the synchronized measurement.

The QUAAC which uses atmospheric products from geostationary satellite to support atmospheric correction of HSR satellite images not only solves the problem of aerosol data acquisition, but it also provides an accurate and fast running atmospheric correction idea for the same type of HSR satellite images. QUAAC is a fully automatic and effective method with good generality. A more accurate inversion of atmospheric parameters [34] will be more conducive to supporting atmospheric correction of HSR remote sensing images.

## Figures and Tables

**Table 1:** Spectral bands of the GF-2 satellite.

Load	Band	Band Range	Spatial Resolution
Panchromatic and Multispectral Camera	1	0.45 µm–0.90 µm	1 m
	2	0.45 µm–0.52 µm	4 m
	3	0.52 µm–0.59 µm	
	4	0.63 µm–0.69 µm	
	5	0.77 µm–0.89 µm	

**Table 2:** Three AOT_uncertainty value ranges correspond to three confidence levels of very good, good, and unreliable.

AOT_Uncertainty (*t*)	Confidence Level
t ≤ 0.5	Very good
0.5<t<1	Good
t ≥ 1	NO_Conf

**Table 3:** The central longitude and latitude, date, and synchronized DEM value and AOD of the five test sites.

Location	Longitude and Latitude	Data	AOD	DEM Value (km)
Dongting Lake	E113.5, N29.2	11 November 2020	0.042	0.140
	E112.2, N29.2	15 January 2021	0.700	0.028
	E113.0, N29.4	23 April 2021	0.233	0.028
Qiyang	E112.0, N26.5	3 September 2021	0.052	0.296
	E111.8, N26.7	6 July 2021	0.175	0.226
Guyuan	E116.0, N41.7	10 August 2020	0.050	1.488
Guangzhou	E113.2, N23.5	29 January 2021	0.142	0.141
Xilinhot	E115.5, N45.2	27 June 2020	0.019	1.277
	E115.1, N44.6	6 August 2020	0.098	1.176
	E116.8, N43.1	16 November 2020	0.0326	1.303

**Table 4:** Information entropy and average gradient of TOA radiance images, QUAAC−corrected images, and FLAASH−corrected images.

Location	TOA Radiance Image EI	QUAAC EI	FLAASH EI	TOA Radiance Image AG	QUAAC AG	FLAASH AG
Dongting Lake	0.109	1.642	2.220	10.55	133.14	88.85
	0.141	1.631	1.790	4.45	46.13	28.63
	0.003	1.563	1.270	4.63	122.86	45.74
Qiyang	0.294	1.530	1.730	12.36	128.77	79.69
	0.532	1.349	1.902	11.86	117.06	76.94
Guyuan	0.175	1.282	1.530	9.24	92.49	55.79
Guangzhou	0.621	2.393	2.650	13.50	180.62	111.70
Xilinhot	0.913	1.352	1.730	5.42	42.94	27.78
	0.106	2.656	2.790	8.27	182.83	93.65
	0.153	1.937	2.270	7.25	70.29	43.01

**Table 5:** MAE, RMSE, R, and $R^{2}$ between FLAASH/QUAAC and MSR in the blue, green, red, and near-infrared bands. The FLA-MSR is FLAASH−corrected SR and the MSR, and the QUA-MSR is QUAAC−corrected SR and the MSR.

	Blue		Green		Red		Near-Infrared	
	FLA-MSR	QUA-MSR	FLA-MSR	QUA-MSR	FLA-MSR	QUA-MSR	FLA-MSR	QUA-MSR
MAE	0.022	0.016	0.026	0.020	0.028	0.024	0.031	0.027
RMSE	0.029	0.021	0.032	0.025	0.034	0.030	0.040	0.038
R	0.784	0.893	0.739	0.819	0.825	0.0890	0.960	0.967
$R^{2}$	0.614	0.797	0.545	0.671	0.681	0.792	0.921	0.935

## Data Availability

Data used in the reported studies were obtained from websites as indicated in the text.
